# Relationship between gut microbiota dysbiosis and immune indicator in children with sepsis

**DOI:** 10.1186/s12887-023-04349-8

**Published:** 2023-10-16

**Authors:** Xia Lin, Mohnad Abdalla, Junjie Yang, Lei Liu, Yali Fu, Yanli Zhang, Shuchun Yang, Han Yu, Yongsheng Ge, Sufang Zhang, Guiyun Kang, Wei Dang, Qin Jiang, Ying Wang, Zhongtao Gai

**Affiliations:** 1 Children’s Hospital Affiliated to Shandong University, Jinan, 250022 China; 2 Jinan Children’s Hospital, Jinan, 250022 China; 3 Shandong Provincial Clinical Research Center for Children’s Health and Disease, Jinan, Shandong 250200 China; 4 College of Life Science, Qilu Normal University, Jinan, Shandong 250200 China

**Keywords:** sepsis, Gut microbiota, Immune indicator, Biomarker, Children

## Abstract

Sepsis is a life-threatening multiple-organ injury caused by disordered host immune response to microbial infection. However, the correlation between gut microbiota dysbiosis and immune indicators remains unexplored. To address this gap in knowledge, we carried out 16 S rDNA sequencing, analyzed clinical fecal samples from children with sepsis (n = 30) and control children (n = 25), and obtained immune indicators, including T cell subtypes (CD3^+^, CD3^+^CD4^+^, CD3^+^CD8^+^, and CD4/CD8), NK cells, cytokines (IL-2, IL-4, IL-6, IL-10, TNF-α and IFN-γ), and immunoglobulin indices (IgA, IgE, IgM and IgG). In addition, we analyzed the correlation between gut microbiota dysbiosis and immune indicators, and evaluated the clinical discriminatory power of discovered bacterial biomarkers. We found that children with sepsis exhibited gut bacterial dysbiosis and low alpha diversity. The Spearman’s rank correlation coefficient suggested that *Rhodococcus erythropolis* had a significantly positive correlation with IFN-γ and CD3^*+*^ T cells. *Klebsiella pneumoniae* and *Streptococcus mitis* were significantly correlated with NK cells. *Bacteroides uniformis* was significantly positively correlated with IgM and erythrocyte sedimentation rate, and *Eubacterium eligens* was significantly positively correlated with IL-4 and CD3^+^CD8^+^ T cells. The biomarkers discovered in this study had strong discriminatory power. These changes in the gut microbiome may be closely related to immunologic dysfunction and to the development or exacerbation of sepsis. However, a large sample size is required for verification.

**Supplementary Information:**

The online version contains supplementary material available at 10.1186/s12887-023-04349-8.

## Introduction

Sepsis, a dysregulated immune response to infection resulting in multi-organ damage and even death, is a major public health threat that affects ∼1.2 million children worldwide each year [1, 2]. Sepsis is the most common cause of death in hospitalized patients, especially in intensive care units (ICUs), having a global mortality rate approaching 25% [3]. Despite its high incidence and mortality rates, the mainstays of therapy—antibiotics and supportive care—have not changed significantly for decades [4]. Gut bacterial translocation occurs frequently in immunocompromised patients and causes sepsis [5]. The effects of the gut microbiome on the occurrence and progression of sepsis are potential therapeutic targets for sepsis and have recently attracted considerable interest.

The gut microbiota significantly regulates the development and functions of innate and adaptive immune systems [6], and gut microbiota dysbiosis is a risk factor for sepsis [7, 8]. Altered gut microbiota influence inflammatory responses and increase gut barrier permeability, which could enable the translocation of pathobionts to the systemic circulation and distant organs. Sepsis leads to intestinal hyperpermeability through the upregulation of inflammation, epithelial cell apoptosis and the alteration of microbiome composition [9, 10]. Proinflammatory mediators, such as tumor necrosis factor-α (TNF-α), interleukin (IL)-1β and IL-6, are recognized as important factors for modulating the intestinal barrier function and increasing gut permeability [11–13]. Translocating endotoxins and bacteria could activate systemic inflammation and promote organ dysfunction and failure [5, 14].

Some 16 S rRNA gene sequencing results have indicated that patients with sepsis have low gut microbiome diversity and high relative abundance of pathogenic bacteria, such as *Staphylococcus* spp, *Klebsiella pneumoniae*, and *Escherichia coli*, which are translocated and cause bacteremia [4]. Gloria et al. [15] found that the intestinal microbiota of ICU patients with sepsis was enriched with harmful microbial species that would magnify the disruption of immune homeostasis. Animal research has shown that mice with increased gut microbiome α-diversity have increased chance of surviving from sepsis because of their increased CD4^+^ T cell response [16]. Furthermore, *Proteobacteria*-rich microbiota resulted in T cell-dependent increases in serum immunoglobulin (Ig) A levels and provided protection against polymicrobial sepsis [17]. The murein lipoprotein and lipopolysaccharide (LPS), which are outer membrane components of Gram-negative bacteria in the gut and provide protection against experimental sepsis by mediating the serum levels of IgG and IgM; the mediation process is dependent on T cells and Toll-like receptor 4 on B cells [18, 19]. Additionally, gut microbial products serve as sources of microbe-associated molecular patterns that bind pattern recognition receptors on innate cells, such as monocytes, macrophages, and natural killer (NK) cells [6]. Sepsis severity is positively correlated with the disbalance between pro-and anti-inflammatory responses, which was profoundly associated with the dysbiosis of the gut microbiota. However, the link between immune response and the gut microbiota in children with sepsis is poorly understood.

To investigate this issue, we performed 16 S rDNA sequencing to explore changes in the composition of the gut microbiota and performed analysis to find correlations between the gut microbiota and immune indicators for children with sepsis. The aim of this study was to provide a possible reference for the diagnosis and treatment of sepsis in children.

## Methods

### Subjects and sample collection

A total of 30 children with sepsis were recruited from the pediatric intensive care unit (PICU) in a Children’s Hospital Affiliated to Shandong University from December 2020 to December 2021. The inclusion criteria were as follows: (1) children with sepsis was diagnosed according to the International Consensus Conference on Pediatric Sepsis [20]; (2) newly diagnosed children with sepsis; (3) the stool samples were collected within 24 h of medical treatment; (4) all stool samples gathered before antibiotic exposure.

Pediatric sepsis was defined the systemic inflammatory response syndrome in the presence of suspected or proven infection. The presence of at least two of the following four criteria, one of which must be abnormal temperature or leukocyte count: (1) Core temperature of > 38.5 °C or < 36 °C; (2) Tachycardia, defined as a mean heart rate > 2 SD above normal for age in the absence of external stimulus, chronic drugs, or painful stimuli; or otherwise unexplained persistent elevation over a 0.5- to 4-h time period OR for children < 1 year old: bradycardia, defined as a mean heart rate < 10 th percentile for age in the absence of external vagal stimulus, β-blocker drugs, or congenital heart disease; or otherwise unexplained persistent depression over a 0.5-h time period; (3) Mean respiratory rate > 2 SD above normal for age or mechanical ventilation for an acute process not related to underlying neuromuscular disease or the receipt of general anesthesia; (4) Leukocyte count elevated or depressed for age (not secondary to chemotherapy-induced leukopenia) or > 10% immature neutrophils.

Twenty-five healthy children were included in this study as controls, who matched the children with sepsis in terms of age and sex. The exclusion criteria: (1) the history of septic infection; (2) antibiotics and/or probiotics within 3 months before enrolling; (3) gastrointestinal problem or any other disease; (4) developmental defects. The study protocol was maintained in accordance with the Declaration of Helsinki and was approved by the ethics committee of Children’s Hospital Affiliated to Shandong University (ETYY-2,020,043). Written informed consent and questionnaires were obtained from the children’s parents.

Fecal samples were collected from each participant and stored at − 80 °C before analysis and 250 mg of stool was preserved in a sterile 2 ml tube (Tinygene Biological Company, China).

### DNA extraction and Illumina sequencing

Total DNA extraction from fecal samples (250 mg, wet weight) was performed using a Fast DNA SPIN kit for feces (MP Biomedicals, Santa Ana, CA, USA) according to the manufacturer’s instructions. The V3–V4 hypervariable region was amplified with a universal primer pair 341 F (5′-CCTACGGGNGGCWGCAG-3′) and 805R (5′-GACTACHVGGGTATCTAATCC-3′) [21]. Sequencing was conducted on an Illumina NovaSeq 6000 system (Illumina Inc., San Diego, CA, USA) using the 2 × 250 paired-end mode according to the standard Illumina platform protocols. All sequencing data are available at NODE (http://www.biosino.org/node) with the accession number OEP003851.

### 16 S rRNA gene sequencing data analysis

Samples were sequenced on an Illumina NovaSeq platform according to the manufacturer’s recommendations. We assigned paired-end reads was assigned to samples according to their unique barcodes, and truncated the reads by cutting off the barcodes and primer sequences. The paired-end reads were merged using FLASH [22]. Using fqtrim (v0.94) (https://ccb.jhu.edu/software/fqtrim/index.shtml), we obtained high-quality clean tags through the quality filtering of the raw reads under specific conditions. Chimeric sequences were filtered using V search software (v2.3.4) [23]. In brief, raw sequence data was demultiplexed and DADA2 was employed to denoise sequencing reads for quality control and the identification of amplicon sequence variants (ASVs) via q2-dada2 plugin [24]. After dereplication using DADA2, we obtained feature table and feature sequence. Alpha diversity and beta diversity were calculated by normalized to the same sequences randomly. Then, according to SILVA (release 138) classifier, feature abundance was normalized using the relative abundance of each sample [25].

The indexes of Alpha diversity (Chao1, Shannon, and observed species) were compared using Mann-Whitney U test at the ASV level [26]. All indices in our samples were calculated with QIIME2 (https://qiime2.org). the difference of microbial composition was further characterized by beta diversity and the data were shown as a principal coordinate analysis (PCoA) calculated by QIIME2 [27]. All weighted and unweighted UniFrac-PCoA plots were complemented by the distance matrix of ASV abundance. The graphs were drawn using the R package, and Blast was used for sequence alignment [28]. The feature sequences were annotated with SILVA database (www.arb-silva.de) for each representative sequences. Linear discriminant analysis effect size was introduced for the identification of bacterial biomarkers between children with sepsis and children in the control group [29]. This procedure was performed on the Galaxy web-based interface (http://huttenhower.sph.harvard.edu/galaxy), and nonparametric factorial Kruskal–Wallis rank-sum test was performed, followed by the linear discriminant analysis (LDA) coupled with measurements, for the assessment of the effect size of each differentially abundant taxon [30]. The threshold of the LDA was set at 4 [31]. Other diagrams were implemented using the R package (v3.5.2).

The ability of microbial markers to differentiate between children with sepsis and children in the control was evaluated using the area under the receiver operating characteristic (ROC) curve [32]. We selected the biomarkers of the bacterial species with the highest LDA scores. These biomarkers had significant correlation with clinical immunological indices.

### Clinical laboratory tests

The association of the taxonomic composition of gut microbiota with sepsis and clinical parameters was assessed. The clinical parameters were collected and determined at the clinical laboratory of Children’s Hospital Affiliated to Shandong University. The clinical indices, including hospital length of stay, white blood cell (WBC), erythrocyte sedimentation rate (ESR), neutrophil cell (NC), C-reactive protein (CRP), procalcitonin (PCT), organ damage number (ODN), T cell subtypes (CD3^+^, CD3^+^CD4^+^, CD3^+^CD8^+^, and CD4/CD8), B lymphocyte (BLC), NK cells, IgA, IgM, IgG, and cytokines (IL-2, IL-4, IL-6, IL-10, TNF-α, and interferon-γ). Serum T-cell subtypes (CD3^+^, CD3^+^CD4^+^, CD3^+^CD8^+^, and CD4/CD8) and NK cells were assessed by flow cytometry. IL-2, IL-4, IL-6, IL-10, TNF-α, and interferon (IFN)-γ levels were quantified by adopting immunoluminescence method.

### Statistical analysis

Data was analyzed using GraphPad Prism (version 7; GraphPad Software, San Diego, CA, USA). We conducted two-tailed t-tests to compare demographics (nonparametric test), a two-tailed significance level of *P* < 0.05 was considered statistically significant. We used standard parameters, except the alpha value of the factorial Mann-Whitney U test. Spearman’s rank correlation was used in determining statistical dependence between continuous variables.

## Results

### Characteristics of study participants

We enrolled 30 children with sepsis (sepsis group, 15 boys and 15 girls) and 25 matched typically developing children without sepsis (control group, 12 boys and 13 girls) in the current study. The average age of children in the sepsis and control groups at the time of sample collection was 3.15 (range, 0.16–9) and 4.016 (range, 0.5–10), respectively.

### Differences of Gut Microbial Diversity between Sepsis and Control Children

This study obtained 2,942,246 high-quality reads and classification at an average of 53,495 reads per sample. At 100% similarity level, this study identified 11,821 ASVs in all samples and an average of 215 ASVs per sample.

**Fig. 1:**
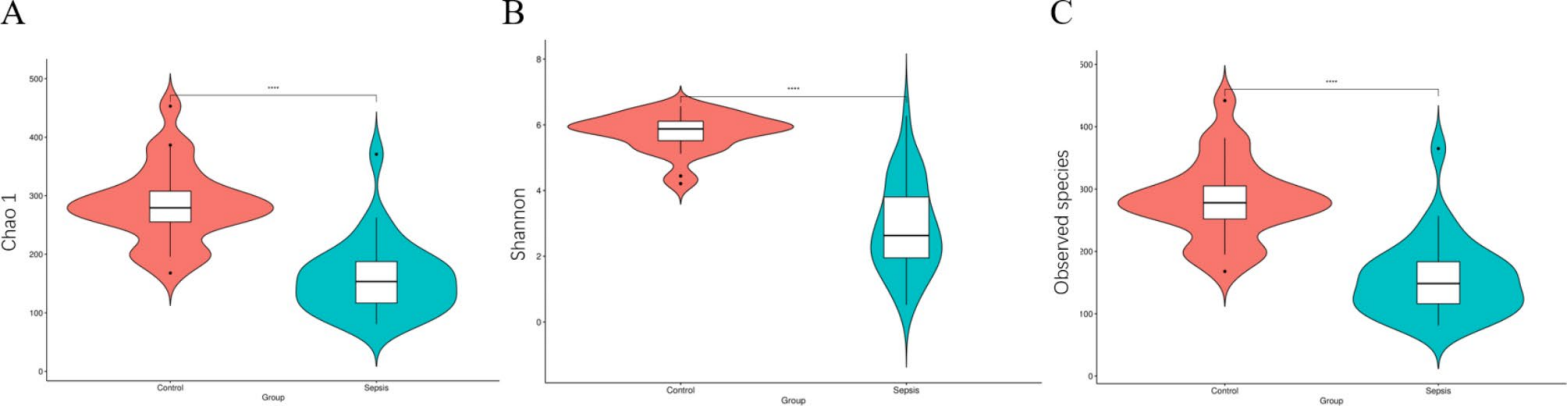
Comparison of α diversity of intestinal microbiota between the sepsis and control groups **A-C**, represent the Chao1, Shannon, Observed species indexes, respectively. *p < 0.05, **p < 0.01

For the diversity characterization of the gut microbiota associated with sepsis, we compared the alpha diversity between the sepsis and control groups. Alpha diversity analysis based on Chao1, Shannon, and observed species that significant decrease in bacterial richness in the sepsis group compared with the control group (Chao1 index 284.41 vs.158.55, *p =* 0.00; Shannon index 5.77 vs. 2.89, *p =* 0.00; observed species index 282.32 vs. 155.53, *p =* 0.00) (Fig. 1).

### Difference in Gut Microbial Composition between Sepsis and Control Groups

**Fig. 2:**
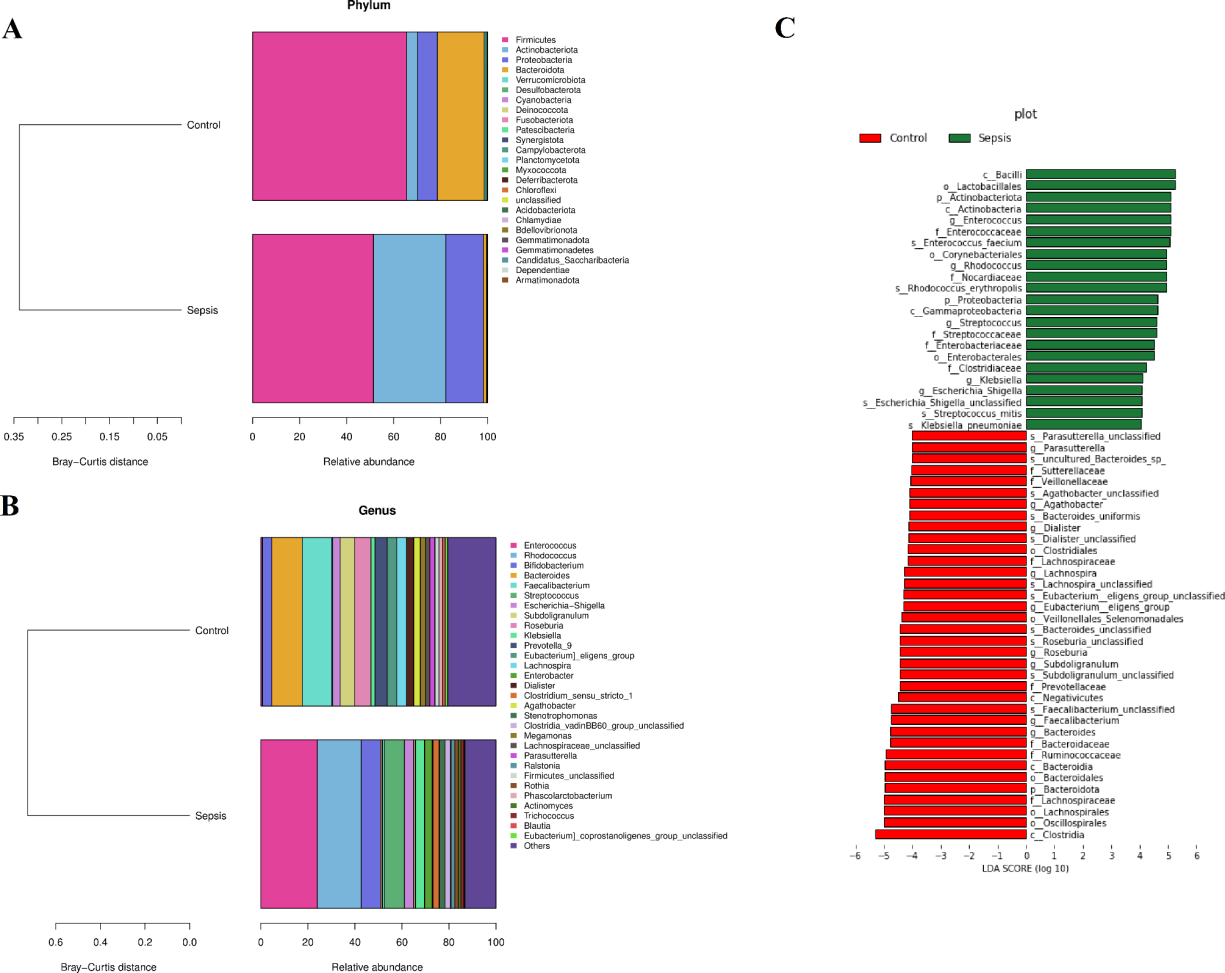
Relative abundance at the phylum level and genus level based on the ASV profile, and LEfSe analysis. **A** and **B**: Relative abundance of different taxa at the phylum level and genus level between sepsis and control groups, respectively. **C**: Histogram of LDA scores computed for differentially abundant taxa between sepsis and control groups. The LDA score indicates the effect size and ranking of each differentially abundant taxon

The relative taxon abundance in the microbiota of both groups were assessed, and the characteristics of gut bacterial immunity in children with sepsis were explored. The total distribution of bacterial taxonomy showed significant variations in bacterial communities between sepsis and control groups at the phylum level (Fig. 2a), as characterized by sharp increase in *Firmicutes/Bacteroidetes* ratio in the children with sepsis (*P* < 0.0001). At the phylum level, significant decrease in the relative abundance of *Firmicutes and Bacteroidetes*, and increase in *Actinobacteria* and *Proteobacteria* were observed in the sepsis group compared with the control group (*P* < 0.01, Fig. 2a). At the genus level, significant increase in the relative abundance of *Enterococcus*, *Rhodococcus*, *Klebsiella*, and *Roseburia*, and a significant reduction in *Bacteroides* and *Faecalibacterium* were observed in the sepsis group compared with the control group (Fig. 2b). Notably, the abundance of *Rhodococcus erythropolis*, *K. pneumoniae*, and *Streptococcus mitis* significantly increased, whereas the abundance of *Bacteroides uniformis* and *Eubacterium eligens* significantly decreased in the children with sepsis, as indicated by the LDA scores (> 4, Fig. 2c).

**Fig. 3:**
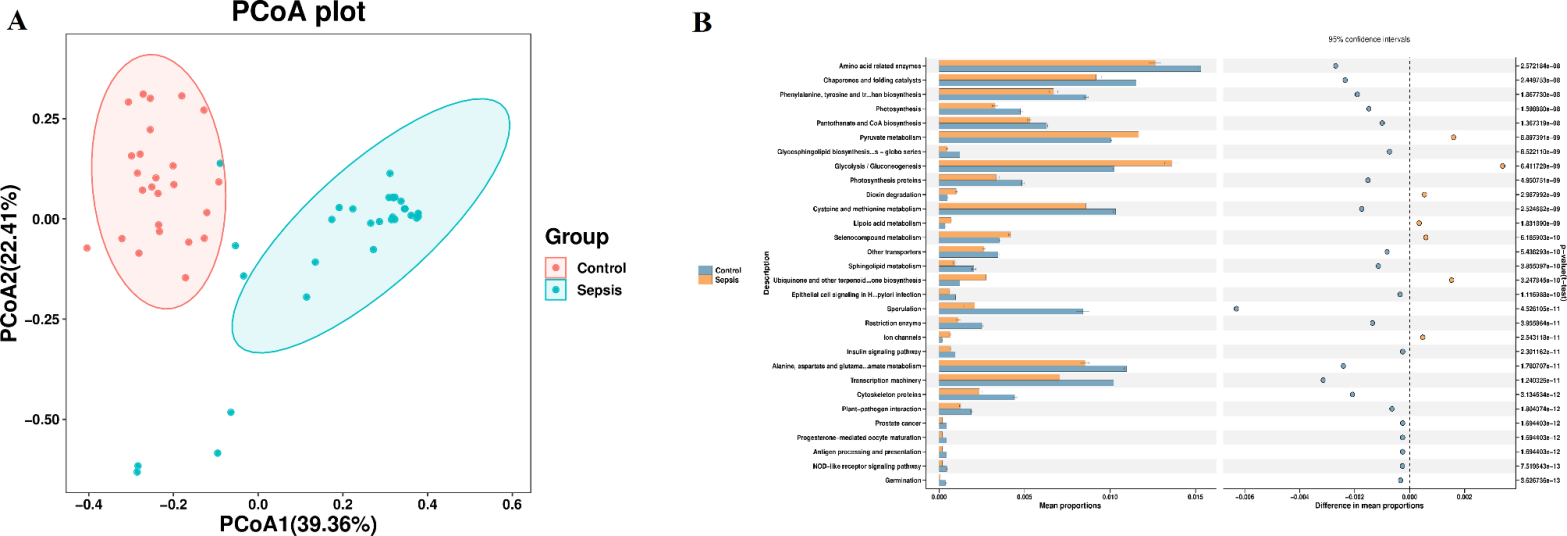
(**A**) PCoA of bacterial beta diversity based on the unweighted UniFrac distance between sepsis and control group. (**B**) Predicted metagenome function based on KEGG pathway analysis

For the β diversity, PCoA data showed that the structure of the gut microbiota in children with sepsis was significantly distinct from that of the children without sepsis (Fig. 3a). Analysis of similarities (ANOSIM) showed significant differences between in children with sepsis and those without sepsis (ANOSIM, r = 0.65, P = 0.001, weighted UniFrac; ANOSIM, r = 0.52, P = 0.001, unweighted UniFrac).

To assess the potential effects of the comorbidities on the microbiota of the children with sepsis, the sepsis samples were divided into three groups according to the number of organ damage (Additional file 1: Table 1): no organ damage (N = 15), one organ damage (N = 7), and multi-organ damage (2 or more organs damage, N = 8). We compared the groups for Shannon index, principal component (PC) 1 and PC2 of the PCoA using Kruskal-Wallis. The co-morbidities did not cause apparent changes in the microbiota in our sepsis cohort (*p* = 0.668), which might be explained by immediate sampling of stool at the beginning of the sepsis (Additional file 2: Table 2).

We investigated phylogenetic communities by reconstructing unobserved states to predict the KEGG pathways [33–35], including pyruvate metabolism, glycolysis/gluconeogenesis, amino acid-related enzymes, chaperones and folding catalysts, phenylalanine, tyrosine and tryptophan biosynthesis signaling pathway showed significant difference between sepsis and control group (Fig. 3b).

### Gut Microbiota and its association with clinical immunological indices

**Fig. 4:**
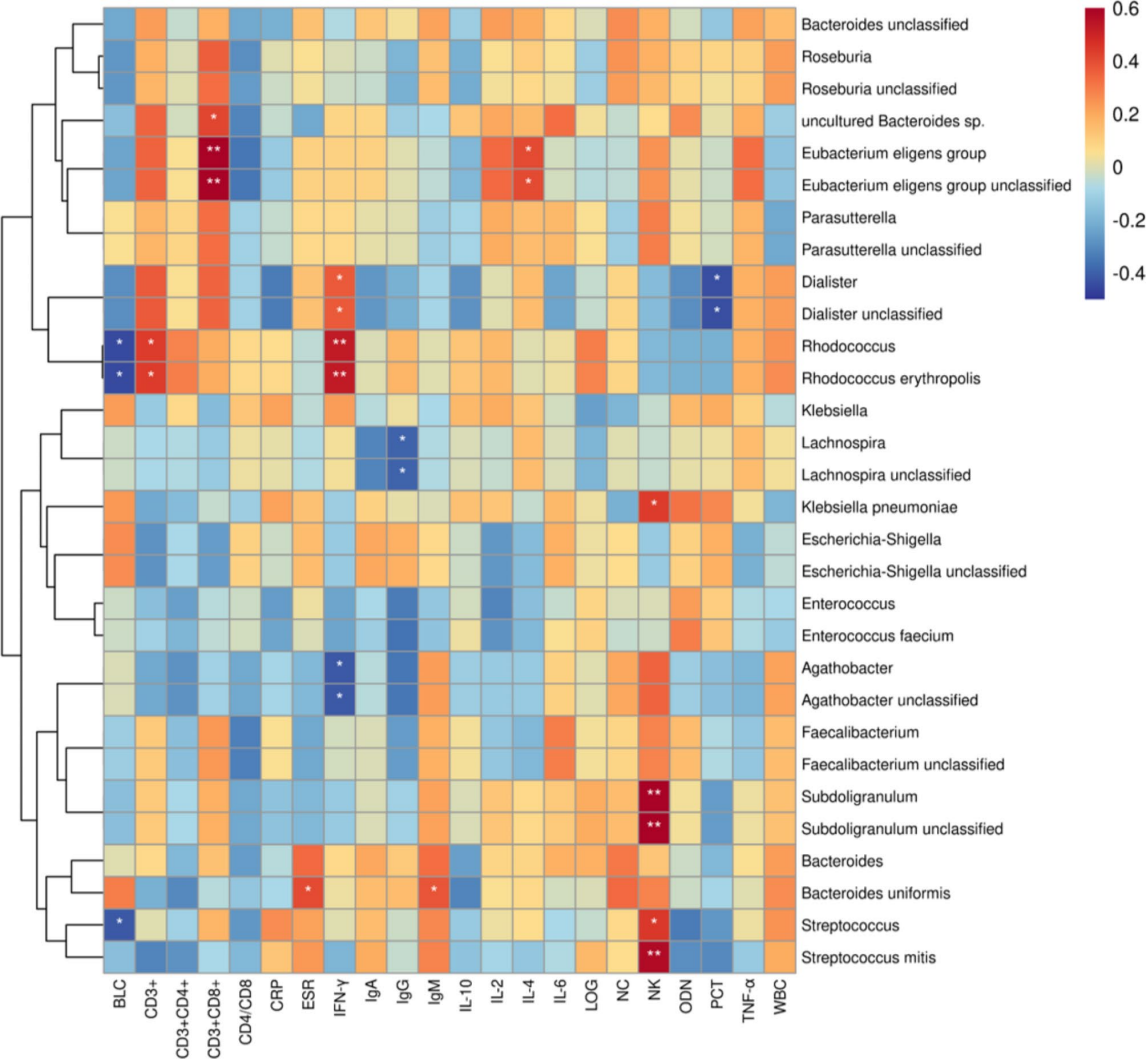
Heatmap of Spearman correlation analysis among the gut microbiota of sepsis and immune indicators. *p < 0.05, **p < 0.01, ***p < 0.001

We calculated Spearman’s rank correlation coefficient of the gut microbiota in children with sepsis (genus and species levels of the differential taxonomy between children with sepsis and healthy children, LDA > 4), several clinical immunological indices and other clinical indices (Fig. 4).

IL-4 showed a significantly positive correlation with *E. eligens.* CD3^+^CD8^+^ T cell was positively correlated with *E. eligens* and uncultured *Bacteroides* sp. NK cells displayed a significantly positive correlation with *K. pneumoniae*, *Subdoligranulum, Streptococcus*, and *Streptococcus mitis*. IgM showed a significantly positive correlation with *B. uniformis*. ESR exhibited a significantly positive correlation with *B. uniformis*. IFN-γ exhibited a significantly positive correlation with *Dialister*, *Rhodococcus*, and *R. erythropolis* and exhibited a significantly negative correlation with *Agathobacter*. IgG and PCT had a significantly negative correlation with *Lachnospira* and *Dialister*, respectively. BLC revealed a significantly negative correlation with *Rhodococcus*, *R. erythropolis*, and *Streptococcus* (Fig. 4).

### Comparison of microbial biomarkers

**Fig. 5:**
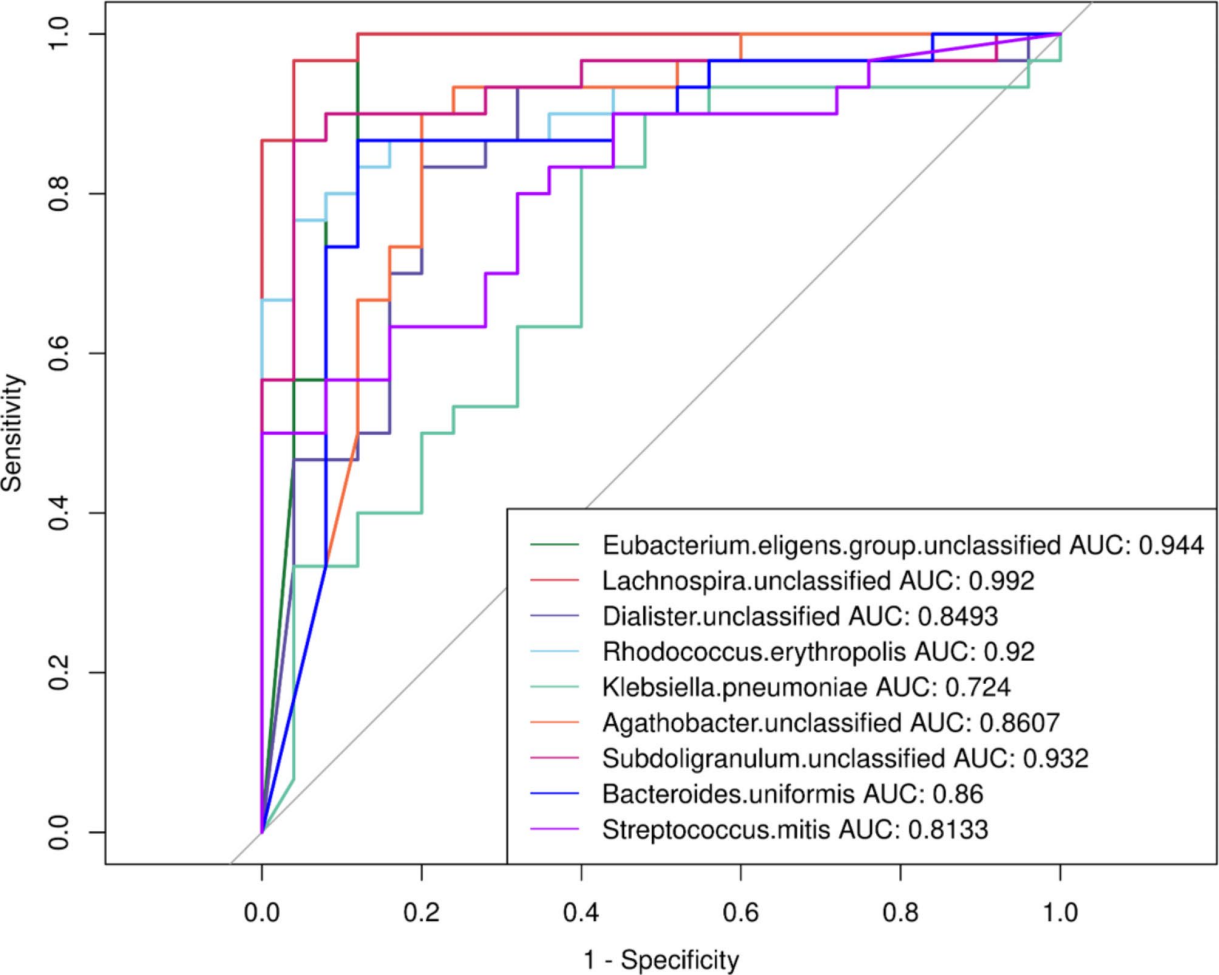
Receiver operating characteristic (ROC) curve of the biomarkers of the bacterial species associated with immunological indices based on the number of bacterial reads. The Area under the curve (AUC) of each ROC curve represents its predictive value regarding a correct classification between sepsis group and control group

To calculate the diagnostic accuracy of bacterial biomarkers, we selected nine biomarkers of the bacterial taxa at the species level. The nine biomarkers had significantly correlation with clinical immunological indices. Out of the nine bacterial species tested, *Lachnospira* showed the best performance in discriminating between sepsis and control group (AUC = 0.992). All of the selected bacterial species with ROC curve (AUC) values were higher than 0.7. The discovered bacterial species with significant correlation with clinical immunological indices may have potential value in diagnosis and risk assessment (Fig. 5).

### The abundant bacterial genus in the gut microbiome corresponds to the pathogen identified by blood culture

7/30 children with sepsis had blood culture-proven sepsis with exclusively single pathogen detection. The blood culture positive cases of the children with sepsis and the corresponding reads in the sequencing data of the stool samples (Additional file 1: Table 1). Due to the limitation of 16s DNA sequencing, the taxon at the species level in the sequencing data were not identified in 4/7 children with sepsis, including *Klebsiella pneumonia, Streptococcus constellatus, and Stenotrophomonas maltophilia.* At the genus level, the pathogen identified by blood culture were all concordant with ASVs detected in the gut microbiota from the children with sepsis. Notably, The pathogen *S. maltophilia* identified by blood culture was the most abundant ASVs in the gut microbiome the most abundant at species and genus levels in case 28.

## Discussion

Sepsis is a heterogeneous and multifaceted dysregulation of the host response to an infecting pathogen in the presence of organ dysfunction [36, 37]. This mode of dysregulation is characterized by simultaneous inflammation and immune suppression. This mode also is affected severely by the dysbiosis of gut microbiota associated with increased risk of bacteremia and microbial translocation [38, 39]. Human and animal studies suggest that the microbiota plays an important role in the pathophysiology of sepsis [40–42]. Our current study is a pilot study to examine the association between the gut bacterial diversity of children with sepsis and clinical immunological indices.

Our findings showed that the structures in the gut microbiome significantly differ between children with sepsis and those without sepsis. Alpha diversity based on Shannon, observed species, and Chao1 indices were significantly reduced in children with sepsis compared with the controls, consistent with the results of Zhanguo Liu et al. [43] and Jing Liu et al. [41]. Gut microbiota dysbiosis with sepsis is possibly due to multifactorial causes, such as antibiotic treatment and massive changes in dietary structure during ICU stays [15]. However, in the current study, a noticeable increase in the relative abundance of common pathogens, including *Enterococcus*, *Klebsiella*, and *Streptococcus*, and significant reduction in *B. uniformis* and *Faecalibacterium* were observed in the sepsis group compared with the control group. The perturbation of the gut microbiome increased bacterial dissemination and might induce or exacerbate systemic inflammation and organ failure [44]. Noteworthy, our results suggested that the microbiome was dominated by *S*. *maltophilia* and was also isolated in blood culture, supporting previously proposed to increase sepsis risk originating from the gut translocation [45].

Notably, the relative abundance of *R. erythropolis* in children with sepsis significantly increased. *R. erythropolis* can cause bloodstream infection and is associated with HIV immunodeficiency [46, 47]. This result indicated that gut microbes translocate to the blood and aggravate microinflammation. The Spearman’s rank correlation coefficients obtained suggested that *R. erythropolis* had a significantly positive correlation with IFN-γ and CD3^*+*^ T cells and a significantly negative correlation with BLC. IFN-γ plays an important role in the pathogenesis of sepsis and exhibits a significantly high plasma levels in patients with sepsis [48, 49]. IFN-γ is considered a potentially valuable biomarker [48]. We proposed that increased abundance of *R. erythropolis*,γ is a risk factor for sepsis and targeting *R. erythropolis* may provide an efficient therapy for sepsis.

Surprisingly, a high abundance of pathogenic bacteria *K. pneumoniae* and *S. mitis* from the fecal samples of the children with sepsis was significantly correlated with NK cells. Previous research conducted on mouse models indicated that NK cells play a vital role in systemic inflammation [50]. Excessive NK cell activation and IFN-γ production can amplify systemic inflammatory response during sepsis and lead to increased physiological dysfunction and risk of death [50, 51]. The overgrowth of *K. pneumoniae* and *S. mitis* from the gut microbiota dysbiosis are closely associated with excessive NK cell activation and IFN-γ production.

Considerably low abundance of *B. uniformis* and *E. eligens* were observed in children with sepsis, providing evidence of reduced fermentation capacity of probiotic bacteria. *B. uniformis* was significantly positively related with IgM and ESR. *E. eligens* was significantly positively correlated with IL-4 and CD3^+^CD8^+^ T cells. Panigrahi et al. reported a randomized, double-blind, and placebo-controlled trial of 4556 infants and showed that a synbiotic containing *Lactobacillus plantarum* can result in 40% relative risk reduction for low respiratory tract infections, death and effectively prevent neonatal sepsis [52]. Studies on animals and humans have shown that probiotics can exert a pivotal effect on the regulation of immune and inflammatory mechanism. Probiotics could control the balance between proinflammatory and anti-inflammatory cytokines and maintain host immune homeostasis [53, 54]. Probiotics improve the differentiation of T-cells against Th2 and development of Th2 cytokines, such as IL-4 and IL-10 [54].

Our study provided evidence that the profitable bacteria are closely related to clinical immunological indices. It can be concluded that administration of probiotics may have a beneficial effect that alleviates sepsis by reducing symptom severity and morbidity through immune homeostasis regulation. However, the main side effects of probiotics are related to bacteremia/fungemia, with predilection found in premature newborns, elderly, immunosuppressed or critically ill patients with severe or fatal comorbidities, or patients in intensive care units treated with broad-spectrum antibiotics on central venous catheters [55]. Thus, there are still some risks and challenges that cannot be ignored in the application of probiotics in children with sepsis.

In the current study, the AUC values of the discovered bacterial species, which had significant correlation with clinical immunological indices, were higher than 0.7. The identified bacterial biomarkers exhibited remarkable discriminatory power for differentiating children with sepsis from healthy control children, demonstrating diagnosis and predictive power for sepsis.

Several important limitations of this study should be highlighted. First, the sample size was small. This study included the children with sepsis hospitalized in only one center. Especially, the gut bacteria biomarkers were required validation in further studies with large multi-center samples. Furthermore, it is necessary to determine the correlation between the metabolites of the gut bacteria and sepsis using multi-omics approach. Finally, the role of intestinal pathogenic bacteria in sepsis were only using correlation analysis in this study. The further studies need to be conducted in cell and animal experiments to identify the causal relationship between gut microbiota and sepsis.

## Conclusion

In summary, we provided unprecedented evidence of the close correlation between disordered gut microbiota and blood immune indicators. Gut microbiota dysbiosis may be an important target for sepsis prevention and therapy. Furthermore, we demonstrated that intestinal bacteria associated with immune indicators are highly sensitive and specific markers that can be used in distinguishing between children with sepsis and healthy children. The discriminatory power of these candidate biomarkers paves the way for establishing fecal microbiome tests for clinical diagnostic and prognostic screening of sepsis. However, a large sample size is required for verification.

### Electronic supplementary material

Below is the link to the electronic supplementary material.

**Additional file 1 Table 1**: The damaged organs and bacterial isolation in the blood culture of all children with sepsis

**Additional file 2 Table 2**: Organ damage influencing the gut microbiome of children with sepsis

## Data Availability

The datasets generated during the current study are available in the NODE repository with the accession number OEP003851 (http://www.biosino.org/node/project/detail/OEP003851).
