# Supplementary material for: Relationship between gut microbiota dysbiosis and immune indicator in children with sepsis
Source: BMC Pediatr. 2023 Oct 16;23:516. doi: 10.1186/s12887-023-04349-8 (PMC10578006; doi:10.1186/s12887-023-04349-8)
Supplement: Supplementary file 1 — Additional file 1 Table 1: The damaged organs and bacterial isolation in the blood culture of all children with sepsis [file 12887_2023_4349_MOESM1_ESM.docx]

**Additional file 1 Table 1: The damaged organs and bacterial isolation in the blood culture of all children with sepsis.**

| No. | The number of organ damage | Damaged organ | The bacteria isolated from blood cultures | Relative abundance [%] of the correspondingly classified sequencing reads at the species level from fecal samples | Relative abundance [%] of the correspondingly classified sequencing reads at the genus level from fecal samples |
| --- | --- | --- | --- | --- | --- |
| 1 | 1 | colon | *—* | *—* | *—* |
| 2 | 7 | heart, lung, liver, stomach, kidneys, brain, blood | *Klebsiella pneumoniae* | 0.06 | 0.36 |
| 3 | 3 | lung, brain, stomach | *—* | *—* | *—* |
| 4 | 0 | — | *—* | *—* | *—* |
| 5 | 0 | — | *—* | *—* | *—* |
| 6 | 0 | — | *—* | *—* | *—* |
| 7 | 0 | — | *—* | *—* | *—* |
| 8 | 0 | — | *Staphylococcus aureus* | *—* | 0.03 |
| 9 | 0 | — | *—* | *—* | *—* |
| 10 | 3 | lung, blood, liver | *—* | *—* | *—* |
| 11 | 0 | — | *—* | *—* | *—* |
| 12 | 5 | liver, heart, kidneys, stomach, blood | *—* | *—* | *—* |
| 13 | 2 | heart, blood | *—* | *—* | *—* |
| 14 | 1 | stomach | *—* | *—* | *—* |
| 15 | 0 | — | *Staphylococcus aureus* | *—* | 0.25 |
| 16 | 1 | lung | *Streptococcus constellatus* | 0.09 | 3.05 |
| 17 | 0 | — | *—* | *—* | *—* |
| 18 | 0 | — | *—* | *—* | *—* |
| 19 | 0 | — | *—* | *—* | *—* |
| 20 | 0 | — | *—* | *—* | *—* |
| 21 | 0 | — | *—* | *—* | *—* |
| 22 | 0 | — | *—* | *—* | *—* |
| 23 | 2 | heart, lung | *—* | *—* | *—* |
| 24 | 1 | colon | *—* | *—* | *—* |
| 25 | 1 | blood | *Escherichia coli* | *—* | 0.25 |
| 26 | 1 | brain | *Streptococcus pneumoniae* | *—* | 7.3 |
| 27 | 3 | heart, liver, blood | *—* | *—* | *—* |
| 28 | 1 | kidneys | *Stenotrophomonas maltophilia* | 65.16 | 65.16 |
| 29 | 0 | — | *—* | *—* | *—* |
| 30 | 3 | heart, kidneys, brain | *—* | *—* | *—* |
