# Supplementary material for: Relationship between gut microbiota dysbiosis and immune indicator in children with sepsis
Source: BMC Pediatr. 2023 Oct 16;23:516. doi: 10.1186/s12887-023-04349-8 (PMC10578006; doi:10.1186/s12887-023-04349-8)
Supplement: Supplementary file 2 — Additional file 2 Table 2: Organ damage influencing the gut microbiome of children with sepsis [file 12887_2023_4349_MOESM2_ESM.docx]

**Additional file 2 Table 2: Organ damage influencing the gut microbiome of children with sepsis.**

| Groups | Sample size | Shannon diversity | | PC1^a^ | PC2 |
| --- | --- | --- | --- | --- | --- |
|  |  |  |  |  |  |
|  |  | Mean±SD^b^ | P^c^ | P (anosim) | |
| no organ damage | N=15 | 2.77±1.58 | 0.23 | 0.88 | 0.085 |
| one organ damage | N=7 | 3.60±1.07 |  | 0.668 | |
| multi-organ damage  (2 or more organs damage) | N=8 | 2.48±0.97 |  |  |  |

a.PC1 and PC2 represent the two most informative principal coordinates (PCs) of the principal coordinate analysis (PCoA), demonstrating the gut microbiota or metabolic structure of sepsis and HC samples. b. SD, standard deviation; c. The difference between two groups were identified by Kruskal-Wallis.
